# Comparing IOL refraction prediction accuracy and A-constant optimization for cataract surgery patients across South Indian and Midwestern United States populations

**DOI:** 10.1186/s12886-025-04217-2

**Published:** 2025-07-02

**Authors:** Omer Siddiqui, Elisa Warner, Miles Greenwald, Tingyang Li, Karthik Srinivasan, Aravind Haripriya, Nambi Nallasamy

**Affiliations:** 1 Department of Ophthalmology and Visual Sciences, Kellogg Eye Center, University of Michigan, 1000 Wall St, Ann Arbor, MI 48105 USA; 2 Department of Computational Medicine and Bioinformatics, University of Michigan, Ann Arbor, MI USA; 3 Cataract and IOL Services, Aravind Eye Hospital, Chennai, India

**Keywords:** Cataract surgery, IOL, Refractive outcomes, Machine learning

## Abstract

**Background:**

IOL power selection is a key determinant of refractive outcomes after cataract surgery. Numerous formulas exist to aid in this process; some are derived from geometric-optical principles (e.g., SRK/T, Barrett) while others are based on data-driven and machine learning approaches (e.g., Nallasamy, Pearl-DGS). Given differences in ocular biometry and environmental stimuli, population-specific factors may impact the generalizability of certain formulas. This study compares clinical and biometric characteristics and evaluates the prediction accuracy of seven IOL power formulas, including machine learning–based approaches, in two distinct cataract surgery populations from South India and the Midwestern United States.

**Methods:**

In this retrospective cross-sectional comparative study, data were collected from two tertiary care eye centers: University of Michigan’s Kellogg Eye Center (Ann Arbor, MI, USA) and Aravind Eye Hospital (Chennai, Tamil Nadu, India). The dataset included demographics, biometry power of the surgically implanted intraocular lens (IOL), and 1-month postoperative refraction. Seven IOL formulas were applied to predict postoperative refraction, and performance was assessed by comparing mean absolute errors both before and after population-specific A-constant optimization.

**Results:**

A total of 985 eyes from Aravind (mean age 60.5 ± 9.5 years) and 1003 from UMich (mean age 70.7 ± 9.5) were analyzed. Aravind patients had significantly lower age, axial length, lens thickness, and central corneal thickness, while UMich patients had lower K measurements, IOL power, and postoperative refraction. Overall, formulas performed better in Aravind for the SN60WF lens. Before A-constant optimization on the Aravind dataset, one formula (Nallasamy) achieved mean absolute error under 0.25 diopters compared to four formulas (Nallasamy, Pearl-DGS, SRK/T, Barrett) afterwards.

**Conclusions:**

Substantial clinical and biometric differences exist between South Indian and Midwestern US cataract populations. Machine learning-based IOL refraction prediction formulas performed the best on the South Indian dataset both before and after population-specific parameter optimization. Understanding population level differences and creating methods to integrate these factors into IOL formulas may help improve refractive outcomes in cataract surgery.

**Supplementary Information:**

The online version contains supplementary material available at 10.1186/s12886-025-04217-2.

## Background

Cataracts cause visual impairment or blindness in over 94 million people worldwide and are the most common cause of blindness [1, 2]. Cataracts are characterized by a gradual loss of transparency in the crystalline lens of the eye, which results in loss of visual acuity. Definitive treatment is achieved through surgically replacing the opacified lens with a permanent artificial intraocular lens (IOL). The power of the implanted IOL is selected to try to achieve a target postoperative refraction. Deviation from the intended target reduces patient satisfaction and can lead to lasting visual disability. Additionally, large refractive errors may necessitate further procedures.

Numerous formulas exist to aid IOL power selection, employing clinical and biometric data to predict post-operative refraction for a given IOL power used. They can be data-driven (regression or machine learning), geometric-optical, or hybrid [3]. Most use a parameter known as the “A-constant” (or other names depending on the formula, such as “lens constant,” “lens factor,” or “surgeon factor”) to capture unaccounted variance due to lens type, population differences, or surgeon-specific factors. In practice, this parameter is empirically determined given specific datasets.

The generalizability of IOL power formulas plays a key role in refractive outcomes after cataract surgeries in different patient populations across the world. Population factors such as genetics, eye shape, nutrition, and UV exposure are hypothesized to cause differences in refraction prediction accuracy [4, 5]. One study has found a higher axial length to corneal curvature (AL/CR) ratio in a Japanese population than reported in other countries, along with a negative correlation between AL/CR ratio and accuracy for certain IOL formulas [6]. A review of 26 population-based studies found that the proportion of post-cataract surgery visual acuity ≥ 0.32 (20/60) was consistently over 70% in studies done in high-income countries, whereas it ranged from 29.9 to 80.5% in low-income and middle-income countries. The review also found refractive error to be one of the leading causes of postoperative visual impairment [7]. Though many factors can play a role in refractive error, IOL formula error is one of them.

The A-constant is an opaque value, representing a “fudge factor” conceptually coming from a variety of unmeasured state variables. Due to this comingling, new data is required to select a lens constant for any change in the context, including a change in population. In practice, collecting the relevant data is not always done, and providers may fall back on parameters selected in other contexts (e.g., populations on which formulas were originally empirically refined or trained). Finding ways to explicitly encode lens and population-level factors to include them into IOL power formulas could theoretically improve generalizability without having to refit parameters to new contexts.

Even with an A-constant chosen for each population separately, differences in accuracy across populations may persist. The functional form of each formula with an empirical component was developed with a certain population and may not perform as well on populations with different underlying factors, such as eye anatomy. Changing the A-constant effectively recenters the output values to aim for zero mean error, but it has no mechanism for encoding different underlying dynamics. We hypothesize that continued learning on new populations is likely to be a valuable feature of machine learning based IOL formulas that have more internal degrees of freedom to learn higher order patterns that improve generalizability.

In this study, we explore two obtained datasets from different populations of patients who underwent cataract surgery, one from the Midwestern United States and another from South India. These populations likely represent differences in genetic and environmental factors. We compare the distributions of preoperative biometric measurements along with IOL prediction accuracy utilizing a variety of formulas. The aim is to better understand the generalizability of existing IOL formulas with a view toward developing formulas that can capture population- and lens-level differences.

## Methods

### Data collection

The study was approved by the Indian Health Service Institutional Review Board (RET202100362) and by the Institutional Review Board at the University of Michigan (HUM00160950). Due to the retrospective and de-identified nature of the data utilized, it was determined by the institutional review boards that informed consent was not required. All research was carried out in accordance with the Declaration of Helsinki.

Data from the South Indian population (“Aravind”) were collected from patients undergoing cataract surgery at Aravind Eye Hospital in Chennai, Tamil Nadu, India between 01/2018 and 06/2022. Preoperative biometry was obtained using Lenstar LS900 optical biometers (Haag-Streit USA, software Lenstar software V.i9.5.0.0). Demographics (patient age, gender, and ethnicity), cataract surgery data, and postoperative refractions were obtained from the Aravind Eye Hospital electronic medical record. Patients included received the Acrysof SN60WF lens (Alcon, Fort Worth, TX, USA).

Data from the Michigan population (“UMich”) were obtained from patients undergoing cataract surgery at University of Michigan’s Kellogg Eye Center between 08/2015 and 06/2019. Preoperative biometry was obtained using Lenstar LS 900 optical biometers (Haag-Streit USA, EyeSuite software V.i9.1.0.0). Demographics (patient age, gender, and ethnicity), cataract surgery data, and postoperative refractions were obtained via the Sight Outcomes Research Collaborative (SOURCE) Ophthalmology Data Repository. Patients included received the Acrysof SN60WF lens (Alcon, Fort Worth, TX, USA).

Data collected for each patient across both populations included age at surgery, sex, biometry measures (anterior chamber depth [ACD], axial length [AL], keratometry of the flat and steep meridians [K1 and K2, respectively], central corneal thickness [CCT], lens thickness [LT], white-to-white distance [WTW]), model and power of the surgically-implanted intraocular lens (IOL), and 1-month postoperative refraction. Manifest refractions at both institutions were performed by trained technicians at the end of the first postoperative month. The inclusion criteria for the cases at both institutions were as follows: (1) Cataract surgery using temporal clear corneal phacoemulsification was performed; (2) A SN60WF IOL was surgically implanted during the cataract surgery; (3) No refractive surgery was performed before the cataract surgery; (4) No additional surgery was performed at the time of cataract surgery. The exclusion criteria were as follows: (1) Incomplete or out-of-bounds data for any of the formulas with which performance was compared; (2) Postoperative best spectacle-corrected visual activity (BSCVA) worse than 20/40; (3) Second eye of the same participant already included to avoid inter-eye correlation.

### IOL power prediction

IOL power prediction was performed using a collection of geometrical optics-based, regression-based, and machine learning-based formulas. These formulas included Barrett Universal II, Haigis, HofferQ, Holladay 1, Nallasamy, PearlDGS, and SRK/T [8–14]. Of these formulas, Barrett, Haigis, HofferQ, Holladay 1, and SRK/T are optics-based, Nallasamy is data-driven, and PearlDGS is a hybrid optics- and machine learned-based formula [3]. The formulas for Haigis, Hoffer Q, Holladay 1, and SRK/T were implemented in Python based on their published equations and updates. The calculations were confirmed with those obtained from Haag-Streit USA EyeSuite software V.i9.1.0.0. Prediction results for Barrett Universal II and Pearl-DGS were obtained through their online calculators. The Nallasamy formula was applied using the original Python implementation [12]. Each formula was used to compute a predicted refraction for the given IOL power. The measured refraction was subtracted to give the refraction prediction error.

The Nallasamy formula was developed using a separate perioperative cataract surgery dataset from the University of Michigan [12]. To avoid any information leakage, no patients included in the model development process for the Nallasamy formula were included in the UMich dataset considered here.

### A-constant optimization

For the Barrett, Haigis, HofferQ, Holladay1, PearlDGS, and SRK/T formulas, the initial A-constants used were the optimized constants from 4013 patients implanted with the SN60WF lens in our previous work [12]. The optimal lens constant for each formula was determined through an empirical optimization process to zero out the mean prediction error. The optimized lens constants were as follows: Barrett: 1.94, Haigis: −0.739, HofferQ: 5.727, Holladay: 1.860, PearlDGS: 119.1, SRK/T: 119.082. The Nallasamy formula does not have a lens constant, and it was used with the model weights as developed in our previous work.

An additional analysis using formula parameters optimized on the Aravind dataset was performed. The Aravind data was split randomly into two groups: 70% in the training set (*N* = 985) and the remaining 30% in the test set (*N* = 296) held out for evaluation purposes. The A-constants for Haigis, HofferQ, Holladay1, Pearl-DGS, and SRK/T were fit using the same process as above to zero out mean prediction error on the 70% training set. To assess Barrett, mean error in the training set was subtracted from each prediction in the test set in order to simulate a lens constant with zero mean absolute error on the training set [15]. For the Nallasamy Formula, the 70% training set was used to perform transfer learning on the original model.

### Statistical analysis

Statistical tests and figures were conducted in Python 3.12 using pandas, scipy, and matplotlib. Differences in demographic and biometric means between the Aravind and UMich datasets were compared two-sided Student *t*-tests.

For the predictive models, the three contexts to analyze performance were the UMich dataset with the formulas using the precomputed A-constants, the Aravind datasets with the formulas using the precomputed A-constants, and the Aravind dataset with the formulas using the Aravind optimized A-constants. In each context, the percentage of eyes within different thresholds of absolute error was computed for each formula. Additionally, the root mean square absolute error (RMSAE), mean absolute error (MAE), median absolute error (MedAE), and standard deviation (SD) of refraction prediction error were computed [15]. RMSAE was included given its use in recent studies comparing IOL performance [16, 17]. Two-sided Student *t*-tests were used to compare the difference between the absolute errors on the Aravind dataset when using precomputed A-constants vs. Aravind optimized A-constants. Performance across IOL formulas was assessed for statistical significance with the Friedman test and Wilcoxon signed-rank tests with Bonferroni correction, applied to the set of absolute errors.

All performance analysis of the Aravind dataset with the formulas using the Aravind optimized A-constants was computed using the held-out testing set, which consisted of a random 30% split of the data not used when optimizing the A-constants. This was done to avoid information leakage and overfit statistics.

## Results

The Aravind dataset included 985 eyes of 985 patients (F = 417, M = 568). The UMich dataset included 1003 eyes of 1003 patients (M = 433, F = 570).

**Fig. 1:**
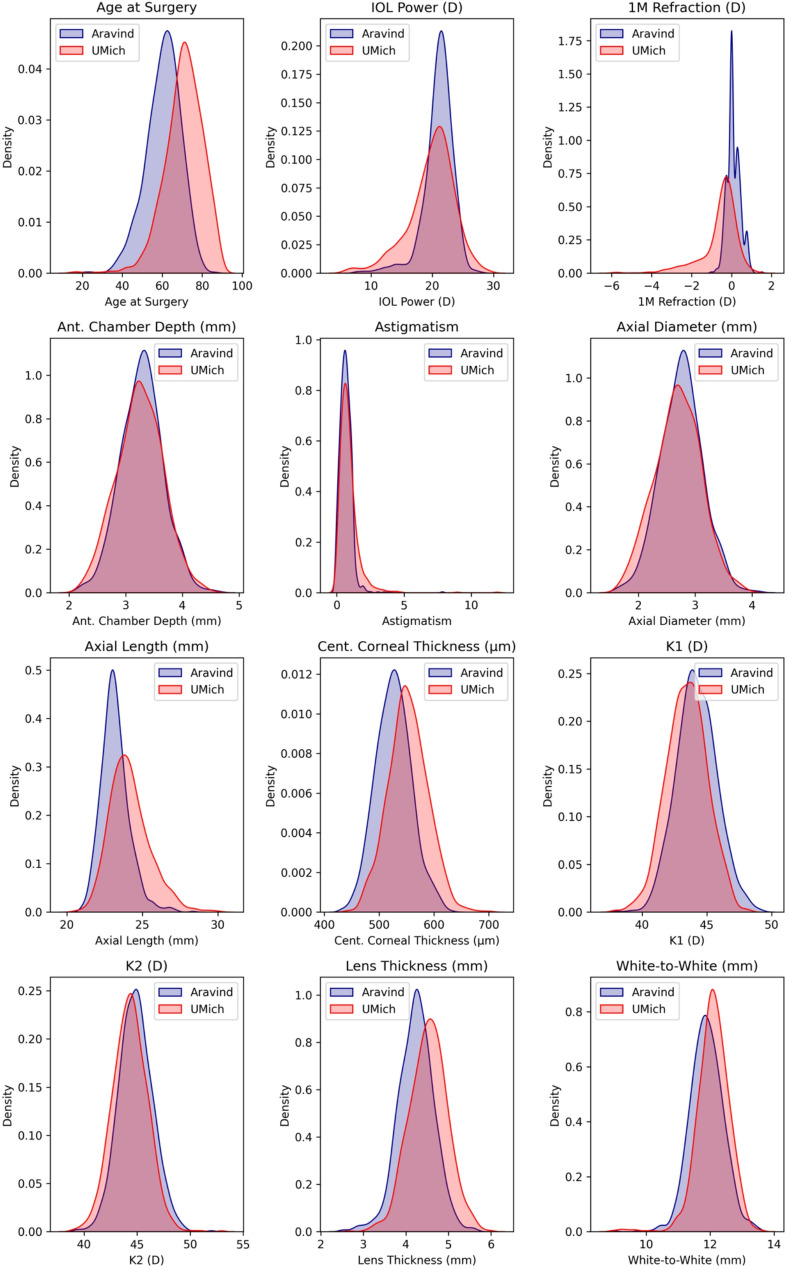
Distributions of patient demographics and eye measurements (Toric removed)

Patient age and biometry measures are summarized for the Aravind and UMich datasets in Table 1. The distributions of these variables across populations are depicted in Fig. 1. Compared to the Aravind group, the UMich population demonstrated significantly lower average IOL power (19.89 D vs. 21.02 D, *p* < 0.01), postoperative refraction (−0.59 vs. 0.10, *p* < 0.01), K1 (43.44 D vs. 44.20 D, *p* < 0.01) and K2 (44.32 D vs. 44.86 D, *p* < 0.01). Compared to the UMich population, the Aravind population demonstrated significantly lower mean age at surgery (60.52 years vs. 70.73 years, *p* < 0.01), axial length (23.19 mm vs. 24.15 mm, *p* < 0.01), and lens thickness (4.24 mm vs. 4.53 mm, *p* < 0.01).

**Table 1:** Patient demographics and eye measurements

	Aravind				UMich				Difference
	Female	Male	All	*p*	Female	Male	All	*p*	*p*
Count (N)	417	568	985	N/A	570	433	1003	N/A	N/A
Laterality (R eye, count)	237	319	556	0.88	283	229	512	0.31	0.02
Age at Surgery	59.50	61.26	60.52	< 0.01	70.92	70.48	70.73	0.47	< 0.01
IOL Power (D)	21.46	20.70	21.02	< 0.01	20.27	19.37	19.89	< 0.01	< 0.01
1 M Refraction (D)	0.03	0.14	0.10	< 0.01	−0.64	−0.51	−0.59	0.02	< 0.01
ACD (mm)	3.22	3.32	3.28	< 0.01	3.19	3.32	3.25	< 0.01	0.05
Astigmatism	0.67	0.66	0.66	0.73	0.85	0.91	0.88	0.26	< 0.01
AD (mm)	2.70	2.79	2.76	< 0.01	2.64	2.77	2.69	< 0.01	< 0.01
AL (mm)	22.91	23.40	23.19	< 0.01	23.93	24.44	24.15	< 0.01	< 0.01
CCT (µm)	522.89	528.51	526.13	< 0.01	550.17	555.74	552.58	0.02	< 0.01
K1 (D)	44.62	43.90	44.20	< 0.01	43.74	43.06	43.44	< 0.01	< 0.01
K2 (D)	45.29	44.56	44.86	< 0.01	44.59	43.97	44.32	< 0.01	< 0.01
Km	44.95	44.23	44.53	< 0.01	44.16	43.51	43.88	< 0.01	< 0.01
LT (mm)	4.20	4.26	4.24	0.03	4.52	4.53	4.53	0.95	< 0.01
WTW (mm)	11.77	11.98	11.89	< 0.01	12.01	12.20	12.09	< 0.01	< 0.01

The *p* values within the Aravind and UMich sub-tables test for significant difference between genders. The *p* values in the last column test for significant difference between institutions

Legend: *IOL* Intraocular lens, *1M Refraction* One-month postoperative refraction, *ACD* Anterior chamber depth, *AD* Axial diameter, *AL* Axial length, *CCT* Central corneal thickness, *LT* Lens thickness, *WTW* White-to-white diameter

The Aravind population showed lower standard deviations in IOL power (2.44 D), refraction (0.33 D), and axial lengths (0.94 mm) compared to the UMich population, whose standard deviations for IOL power, refraction, and AL were 3.78 D, 0.93 D, and 1.35 mm, respectively.

**Fig. 2:**
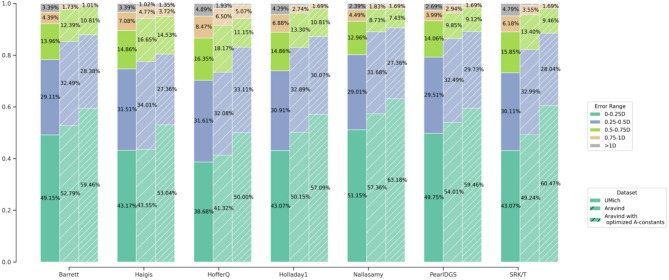
Percent error of IOL Formula by population

Lens constants optimized to the Aravind 70% training set are given in Table S1. The percentage of patients in each error category for each IOL refraction prediction formula is shown in Fig. 2, separately for UMich, Aravind using the UMich A-constants (“unoptimized Aravind”), and Aravind using the Aravind optimized A-constants (“optimized Aravind”). The statistics for optimized Aravind only include the 30% testing set, which was not used when setting the A-constant. For UMich, the three formulas with the highest percentage of eyes under 0.25 diopters of error were Nallasamy (51.15%), PearlDGS (49.75%), and Barrett (49.15%). The corresponding list for unoptimized Aravind is PearlDGS (57.87%), Nallasamy (57.36%), and Barrett (52.79%). All formulas demonstrated better accuracy in the unoptimized Aravind population compared with the UMich population. Performance additionally improved across the board between unoptimized and optimized Aravind. The formulas with the highest percentage of eyes under 0.25 diopters of error for Aravind were Nallasamy (63.18%), SRK/T (60.47%), Barrett (59.46%), and PearlDGS (59.46%). The formulas with the most improved percentages under 0.25 diopters were SRK/T (+ 11.23%), Haigis (+ 9.49%), and HofferQ (+ 8.68%). The least improved were PearlDGS (+ 5.45%), Nallasamy (+ 5.82%), and Barrett (+ 6.67%).

Table 2 shows formula performance with UMich A-constants and optimized A-constants on the Aravind cohort. Before optimizing, the lowest MAEs were observed with Nallasamy (0.25), PearlDGS (0.27), and Barrett (0.28). After optimization (and transfer learning), the lowest MAEs were observed with Nallasamy (0.23), PearlDGS (0.24), Barrett (0.25) and SRK/T (0.25).

As noted, mean absolute errors for Aravind decreased for every formula after optimizing the A-constant to the Aravind data set. The significance of this decreases was the least for Nallasamy and PearlDGS, and it was the most for SRK/T and HofferQ.

**Table 2:** Formula performance on Aravind before and after A-constant optimization

	Aravind with UMich A-constant					Aravind with Aravind optimized A-constant					Difference in AE
	MAE (D)	RMSAE (D)	ME (D)	Std (D)	Within 0.5D (%)	MAE (D)	RMSAE (D)	ME (D)	Std (D)	Within 0.5D (%)	*p*-value
Barrett	0.28	0.35	0.12	0.33	85.28	0.25	0.31	−0.02	0.31	87.84	0.01
Haigis	0.33	0.41	0.15	0.38	77.56	0.29	0.38	−0.03	0.38	80.41	0.02
HofferQ	0.36	0.45	0.19	0.4	73.4	0.29	0.37	−0.02	0.37	83.11	< 0.01
Holladay1	0.3	0.37	0.13	0.35	83.05	0.26	0.33	−0.03	0.33	87.16	0.01
Nallasamy	0.25	0.32	0.01	0.32	89.04	0.23	0.3	−0.02	0.3	90.54	0.10
PearlDGS	0.27	0.35	0.03	0.35	86.5	0.24	0.31	−0.03	0.31	89.19	0.03
SRK/T	0.3	0.37	0.14	0.34	82.23	0.25	0.32	−0.04	0.31	88.51	< 0.01

The *p*-values test for significant difference in the mean absolute error for the Aravind data with UMich A-constants and Aravind data with Aravind optimized A-constants

Legend: *MAE *Mean absolute error, *RMSAE *Root mean square absolute error, *ME *Mean error, *SD *Standard deviation of error, Within 0.5D % = percentage with absolute error within 0.5D

**Fig. 3:**
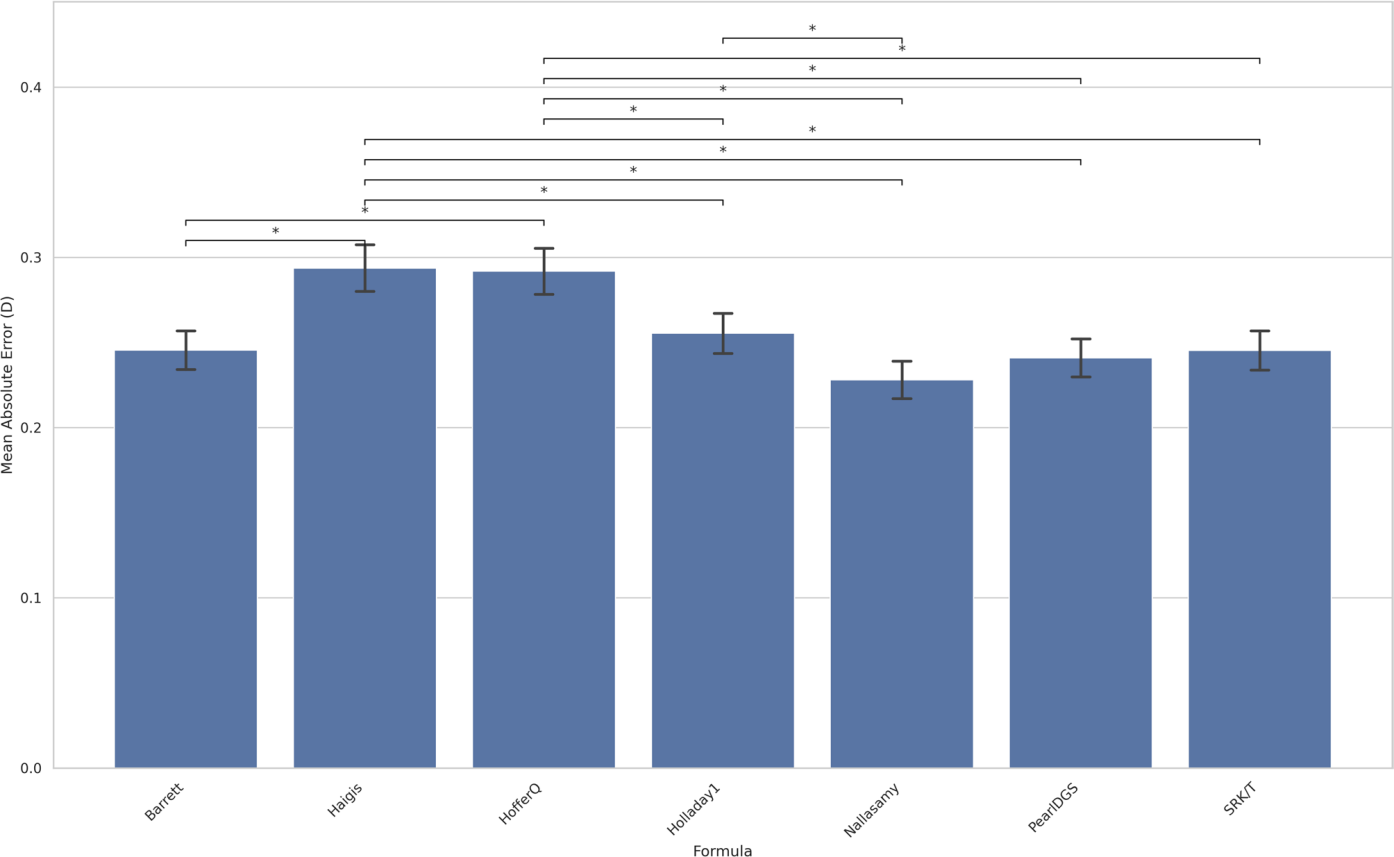
Comparison of formula performance on Aravind with optimized A-constants. Legend: Bars indicate mean absolute error. The error bars show the standard deviation of absolute error. * indicate statistically significant difference between a pair of formulas at the 5% significance level

Figure 3 shows the mean absolute errors by formula for Aravind with optimized Aravind A-constant, along with the pairs of formulas with statistically significant difference in performance.

## Discussion

In this study, optical biometry and refraction prediction performance in two different populations undergoing cataract surgery were compared: (1) A Midwest population in the United States (“UMich”), and (2) A South Indian population (“Aravind”). Though not measured, the populations differ in racial makeup and environmental factors such as diet and UV exposure.

The Aravind cohort demonstrated consistently lower axial lengths compared to the UMich cohort. Given longer axial length correlates with myopia, this finding is consistent with higher reported prevalence of myopia in the American Midwest at 50.1% compared to 43% at Aravind among patients 55–60 years old [18, 19]. The difference in axial length may be explained in part by higher levels of UV exposure, which have been shown to be negatively correlated with axial length [20]. As South India is close to the equator, the yearly level of UV exposure for local residents is higher than for residents of the midwestern United States, where winters are often overcast and sunlight hours are shorter. The younger patient age observed at the time of surgery in the Aravind cohort may be due to earlier onset of cataracts, for which some of the hypothetical contributors include nutrition, diabetes, environmental exposure, or UV exposure.

Central corneal thickness (CCT) and lens thickness were significantly lower in the Aravind cohort compared to the Michigan cohort. In a prior study, a lower CCT was seen in a Japanese population compared to a Caucasian population [21]. The South Indian cohort in this study demonstrated even lower CCTs than the Japanese group. Measurements of the Michigan group and the South Indian group in our study are consistent with findings in other studies [22]. The CCT finding does contradict prior studies suggesting that UV exposure is associated with higher CCT [23]. Since the UMich cohort was older at the time of surgery than the Aravind cohort, and age is correlated with lens thickness, the difference in lens thickness is likely explained in part by the older age of the UMich cohort [24]. Another biometric difference seen was significantly lower K1 and K2 in the UMich cohort compared to the Aravind cohort.

The UMich cohort demonstrated a wider range of postoperative refractions than the Aravind cohort. This difference is consistent with differences in practice regarding refractive target. Aravind Eye Hospital’s standard practice is to target a refraction near 0 (emmetropia) to streamline preoperative planning, leading to lower variance in postoperative refractions.

In the assessment of refraction prediction performance across the two cohorts, all formulas performed better with the Aravind cohort compared to UMich. Previous research has suggested that IOL formula performance is sensitive to axial length [25, 26]. The higher mean axial length, wider range of axial lengths, and skewed distribution of axial lengths within the UMich cohort are likely contributing factors to the poorer refraction prediction performance of all formulas in the UMich cohort. Another possible contributor is the practice patterns regarding target refractions, as the distribution of formula errors may not be consistent across the level of predicted refractions.

On our UMich dataset, Barrett, Nallasamy, and Pearl-DGS were shown to outperform Haigis, Hoffer Q, Holladay1, and SRK/T. The same three performed the best on the Aravind group using the UMich A-constants. Performance after re-optimization on the Aravind group was topped by the three data-driven formulas: Nallasamy, Pearl-DGS, and SRK/T, with Barrett performance slightly under SRK/T. Across all contexts tested, the machine learning-based Nallasamy and Pearl-DGS formulas performed the best. Among optics-based formulas, Barrett consistently performed the best. Though machine learning-based formulas lack in interpretability, the evidence here suggests they outperform traditional optics-based formulas. Furthermore, the machine learning-based formulas considered here managed to generalize to a new dataset despite the risk of overfitting to training data.

As IOL formulas are used in varying geographies, generalizability and accuracy on new populations is a critical piece to cataract surgery outcomes. Optimizing parameters on new populations consistently improved performance for all formulas tested, but the data and computational resource-based barriers needed to carry it out can make it impractical without the resources of a research institution. For this reason, encoding population level variation in the base formula would be ideal. Population-level parameter optimization for the Aravind dataset had the least marginal benefit for machine learning formulas. This finding, along with better performance at baseline, suggests that machine learning based approaches are able to more effectively capture latent variables that differ among populations compared to traditional optics-based formulas.

A limitation is the underrepresentation of longer axial lengths (only 12 of 985 Aravind SN60WF patients). Higher sun exposure may contribute to fewer long axial lengths in South India [20]yet evolving factors—such as increased education [19] and near-work ratios [27] - may lead to higher axial lengths in future generations.

Further work can include other IOL formulas, such as EVO, Hill-RBF, and Kane. This study focused on a single lens across two specific populations, which may limit its applicability. Research incorporating a range of IOLs and populations will help further characterize the generalizability of IOL formulas and dependence on the A-factor.

## Conclusions

In the modern era of data-driven IOL power calculations, IOL formula generalizability is essential to ensure high quality refractive outcomes for populations for which formulas were not originally developed. In this study, we have shown a comparative analysis of a South Indian cohort and a Midwestern United States cohort to demonstrate that distributions of biometric parameters and IOL calculation performance can vary substantially by region of the world. These findings underscore the importance of (1) broadly representative datasets for the development of IOL power calculation tools and (2) developing calculation methods that generalize easily to different populations.

## Supplementary Information

Supplementary Material 1.

## Data Availability

No datasets were generated or analysed during the current study.

## Abbreviations

AE: Absolute error
AL/CR: Axial length to corneal curvature
ACD: Anterior chamber depth
AD: Axial diameter
AL: Axial length
CCT: Central corneal thickness
IOL: Intraocular lens
K1: Keratometry of the flat and steep meridian
K2: Keratometry of the steep meridian
LT: Lens thickness
MAE: Mean absolute error
MedAE: Median absolute error
SD: Standard deviation
WTW: White-to-white distance

## References

[1] Lam D, Rao SK, Ratra V, et al. Cataract. Nat Rev Dis Primers. 2015;1(1):15014. 10.1038/nrdp.2015.14. 27188414 10.1038/nrdp.2015.14

[2] GBD 2019 Blindness and Vision Impairment Collaborators, Vision Loss Expert Group of the Global Burden of Disease Study. Causes of blindness and vision impairment in 2020 and trends over 30 years, and prevalence of avoidable blindness in relation to VISION 2020: the right to sight: an analysis for the global burden of disease study. Lancet Glob Health. 2021;9(2):e144–60. 10.1016/S2214-109X(20)30489-7. 33275949 10.1016/S2214-109X(20)30489-7 PMC7820391

[3] Stopyra W, Langenbucher A, Grzybowski A. Intraocular Lens power calculation Formulas-A systematic review. Ophthalmol Ther. 2023;12(6):2881–902. 10.1007/s40123-023-00799-6. 37698825 10.1007/s40123-023-00799-6 PMC10640516

[4] Tavani A. Food and nutrient intake and risk of cataract. Ann Epidemiol. 1996;6(1):41–6. 10.1016/1047-2797(95)00099-2. 8680624 10.1016/1047-2797(95)00099-2

[5] Ji Y, Cai L, Zheng T, et al. The mechanism of UVB irradiation induced-apoptosis in cataract. Mol Cell Biochem. 2015;401(1–2):87–95. 10.1007/s11010-014-2294-x. 25445170 10.1007/s11010-014-2294-x

[6] Omoto MK, Torii H, Hayashi K, Ayaki M, Tsubota K, Negishi K. Ratio of axial length to corneal radius in Japanese patients and accuracy of intraocular Lens power calculation based on biometric data. Am J Ophthalmol. 2020;218:320–9. 10.1016/j.ajo.2020.03.006. 32209342 10.1016/j.ajo.2020.03.006

[7] Han X, Zhang J, Liu Z, et al. Real-world visual outcomes of cataract surgery based on population-based studies: a systematic review. Br J Ophthalmol. 2023;107(8):1056–65. 10.1136/bjophthalmol-2021-320997. 35410876 10.1136/bjophthalmol-2021-320997 PMC10359559

[8] Barrett GD. An improved universal theoretical formula for intraocular lens power prediction. J Cataract Refract Surg. 1993;19(6):713–20. 10.1016/S0886-3350(13)80339-2. 8271166 10.1016/s0886-3350(13)80339-2

[9] Haigis W. Intraocular lens calculation after refractive surgery for myopia: Haigis-L formula. J Cataract Refract Surg. 2008;34(10):1658–63. 10.1016/j.jcrs.2008.06.029. 18812114 10.1016/j.jcrs.2008.06.029

[10] Hoffer KJ. The hoffer Q formula: A comparison of theoretic and regression formulas. J Cataract Refract Surg. 1993;19(6):700–12. 10.1016/S0886-3350(13)80338-0. 8271165 10.1016/s0886-3350(13)80338-0

[11] Holladay JT, Musgrove KH, Prager TC, Lewis JW, Chandler TY, Ruiz RS. A three-part system for refining intraocular lens power calculations. J Cataract Refract Surg. 1988;14(1):17–24. 10.1016/S0886-3350(88)80059-2. 3339543 10.1016/s0886-3350(88)80059-2

[12] Li T, Stein J, Nallasamy N. Evaluation of the Nallasamy formula: a stacking ensemble machine learning method for refraction prediction in cataract surgery. Br J Ophthalmol. 2023;107(8):1066–71. 10.1136/bjophthalmol-2021-320599. 35379599 10.1136/bjophthalmol-2021-320599 PMC9530066

[13] Debellemanière G, Dubois M, Gauvin M, et al. The PEARL-DGS formula: the development of an Open-source machine Learning–based Thick IOL calculation formula. Am J Ophthalmol. 2021;232:58–69. 10.1016/j.ajo.2021.05.004. 33992611 10.1016/j.ajo.2021.05.004

[14] Retzlaff JA, Sanders DR, Kraff MC. Development of the SRK/T intraocular lens implant power calculation formula. J Cataract Refract Surg. 1990;16(3):333–40. 10.1016/S0886-3350(13)80705-5. 2355321 10.1016/s0886-3350(13)80705-5

[15] Holladay JT, Wilcox RR, Koch DD, Wang L. Review and recommendations for univariate statistical analysis of spherical equivalent prediction error for IOL power calculations. J Cataract Refract Surg. 2021;47(1):65–77. 10.1097/j.jcrs.0000000000000370. 32769751 10.1097/j.jcrs.0000000000000370

[16] Stopyra W, Voytsekhivskyy O, Grzybowski A. Accuracy of 7 artificial Intelligence–Based intraocular Lens power calculation formulas in extremely long Caucasian eyes. Am J Ophthalmol. 2025;271:337–46. 10.1016/j.ajo.2024.10.033. 39536848 10.1016/j.ajo.2024.10.033

[17] Stopyra W, Voytsekhivskyy O, Grzybowski A. Accuracy of 20 intraocular Lens power calculation formulas in Medium-Long eyes. Ophthalmol Ther. 2024;13(7):1893–907. 10.1007/s40123-024-00954-7. 38734806 10.1007/s40123-024-00954-7 PMC11178744

[18] AlMahmoud T, Priest D, Munger R, Jackson WB. Correlation between refractive error, corneal power, and thickness in a large population with a wide range of ametropia. Invest Ophthalmol Vis Sci. 2011;52(3):1235. 10.1167/iovs.10-5449. 21051694 10.1167/iovs.10-5449

[19] Joseph S, Krishnan T, Ravindran RD, et al. Prevalence and risk factors for myopia and other refractive errors in an adult population in Southern India. Ophthalmic Physiologic Optic. 2018;38(3):346–58. 10.1111/opo.12447. 10.1111/opo.12447 PMC6001660 29574882

[20] Read SA, Collins MJ, Vincent SJ. Light exposure and eye growth in childhood. Invest Ophthalmol Vis Sci. 2015;56(11):6779. 10.1167/iovs.14-15978. 26567790 10.1167/iovs.14-15978

[21] Aghaian E, Choe JE, Lin S, Stamper RL. Central corneal thickness of caucasians, chinese, hispanics, filipinos, African americans, and Japanese in a glaucoma clinic. Ophthalmology. 2004;111(12):2211–9. 10.1016/j.ophtha.2004.06.013. 15582076 10.1016/j.ophtha.2004.06.013

[22] Vijaya L, George R, Arvind H, et al. Central corneal thickness in adult South Indians. Ophthalmology. 2010;117(4):700–4. 10.1016/j.ophtha.2009.09.025. 20079536 10.1016/j.ophtha.2009.09.025

[23] Čejka Č, Luyckx J, Čejková J. Central corneal thickness considered an index of corneal hydration of the UVB irradiated rabbit cornea as influenced by UVB absorber. Physiol Res Published Online June. 2012;30:299–306. 10.33549/physiolres.932242. 10.33549/physiolres.932242 22480421

[24] Meng J, Wei L, He W, Qi J, Lu Y, Zhu X. Lens thickness and associated ocular biometric factors among cataract patients in Shanghai. Eye Vis. 2021;8(1):22. 10.1186/s40662-021-00245-3. 10.1186/s40662-021-00245-3 PMC8165789 34053465

[25] Stopyra W. Analysis of accuracy of twelve intraocular lens power calculation formulas for eyes with axial hyperopia. Saudi J Ophthalmol. 2023;37(2):125–30. 10.4103/sjopt.sjopt_64_22. 37492218 10.4103/sjopt.sjopt_64_22 PMC10365256

[26] Stopyra W. Analysis of accuracy of twelve intraocular lens power calculation formulas for eyes with axial myopia. Taiwan J Ophthalmol. 2023;13(2):225–30. 10.4103/2211-5056.357849. 37484619 10.4103/2211-5056.357849 PMC10361426

[27] Gopalakrishnan A, Hussaindeen JR, Sivaraman V, et al. Myopia and its association with near work, outdoor time, and housing type among schoolchildren in South India. Optom Vis Sci. 2023;100(1):105–10. 10.1097/OPX.0000000000001975. 36705720 10.1097/OPX.0000000000001975

