# Supplementary material for: Comparing IOL refraction prediction accuracy and A-constant optimization for cataract surgery patients across South Indian and Midwestern United States populations
Source: BMC Ophthalmol. 2025 Jul 2;25:349. doi: 10.1186/s12886-025-04217-2 (PMC12219035; doi:10.1186/s12886-025-04217-2)
Supplement: Supplementary file 1 — Supplementary Material 1. [file 12886_2025_4217_MOESM1_ESM.docx]

**Table S1: Aravind Optimized A-constants**

| **Formula** | **A-constant** |
| --- | --- |
| **Holladay 1** | **1.749** |
| **SRK/T** | **118.894** |
| **HofferQ** | **5.571** |
| **Haigis** | **-0.862** |
| **PearlDGS** | **119.05** |
